# The Current Treatment Paradigm for Pancreatic Ductal Adenocarcinoma and Barriers to Therapeutic Efficacy

**DOI:** 10.3389/fonc.2021.688377

**Published:** 2021-07-15

**Authors:** Daniel R. Principe, Patrick W. Underwood, Murray Korc, Jose G. Trevino, Hidayatullah G. Munshi, Ajay Rana

**Affiliations:** ^1^ Medical Scientist Training Program, University of Illinois College of Medicine, Chicago, IL, United States; ^2^ Department of Surgery, University of Illinois at Chicago, Chicago, IL, United States; ^3^ Department of Surgery, University of Florida, Gainesville, FL, United States; ^4^ Department of Developmental and Cell Biology, University of California, Irvine, CA, United States; ^5^ Department of Surgery, Division of Surgical Oncology, Virginia Commonwealth University, Richmond, VA, United States; ^6^ Department of Medicine, Northwestern University Feinberg School of Medicine, Chicago, IL, United States; ^7^ Jesse Brown VA Medical Center, Chicago, IL, United States

**Keywords:** pancreatic cancer, chemotherapy, radiation, surgery, immunotherapy, targeted therapy, treatment

## Abstract

Pancreatic ductal adenocarcinoma (PDAC) has a dismal prognosis, with a median survival time of 10-12 months. Clinically, these poor outcomes are attributed to several factors, including late stage at the time of diagnosis impeding resectability, as well as multi-drug resistance. Despite the high prevalence of drug-resistant phenotypes, nearly all patients are offered chemotherapy leading to modest improvements in postoperative survival. However, chemotherapy is all too often associated with toxicity, and many patients elect for palliative care. In cases of inoperable disease, cytotoxic therapies are less efficacious but still carry the same risk of serious adverse effects, and clinical outcomes remain particularly poor. Here we discuss the current state of pancreatic cancer therapy, both surgical and medical, and emerging factors limiting the efficacy of both. Combined, this review highlights an unmet clinical need to improve our understanding of the mechanisms underlying the poor therapeutic responses seen in patients with PDAC, in hopes of increasing drug efficacy, extending patient survival, and improving quality of life.

## Introduction

Pancreatic ductal adenocarcinoma (PDAC) is currently the third leading cause of cancer death in the US. Risk factors for PDAC include tobacco smoking, germline mutations in such genes as breast cancer gene 1 (*BRCA1*) and *BRCA2*, chronic pancreatitis, obesity, long-standing type 2 diabetes (T2DM), and prolonged and excessive alcohol consumption (1–3). There has been a slow but progressive increase in PDAC incidence in the US, but the overall survival rate has also increased. Currently, the overall 5-year survival rate is approximately 10% (4). Based on recent trends in PDAC incidence and survival and the improvements in survival in cancers of the lung and breast, it has been proposed that PDAC will become the second leading cause of cancer-related death by 2030 (5). While surgical resection offers a clear survival benefit and increases 5-year survival to 25%, the majority of patients present with disseminated and/or locally advanced disease, precluding them from undergoing resection. As such, nearly all are offered conventional chemotherapy. While chemotherapy provides a survival benefit in both resectable and non-resectable forms of the disease, these benefits are modest as almost all patients harbor some degree of drug resistance (6). Further, a significant number of patients experience grade 3-4 adverse effects (6).

While several chemotherapy regimens have been approved for metastatic PDAC, the most widely used and best-studied agent is Gemcitabine, a drug was first approved by the FDA for metastatic PDAC in 1996, after showing marginal efficacy in clinical trials (7–9). It has remained in clinical use, often in combination with albumin-bound (Nab) Paclitaxel, which improved survival time compared to Gemcitabine monotherapy (10). The multi-drug regimen FOLFIRINOX (5-Fluorouracil, Leucovorin, Irinotecan, and Oxaliplatin) has also shown efficacy in metastatic PDAC. In fact, FOLFIRINOX offers improved disease-free survival compared to Gemcitabine (21.6 v 12.8 months), though FOLFINIROX is associated with a higher rate of serious adverse effects (75.9 v 52.9%) (11). Despite their spectrum of toxicities, FOLFIRINOX and Gemcitabine with Nab-Paclitaxel remain the best and most widely prescribed medications for patients seeking treatment.

## Standard of Care Treatment Overview

**Figure 1:**
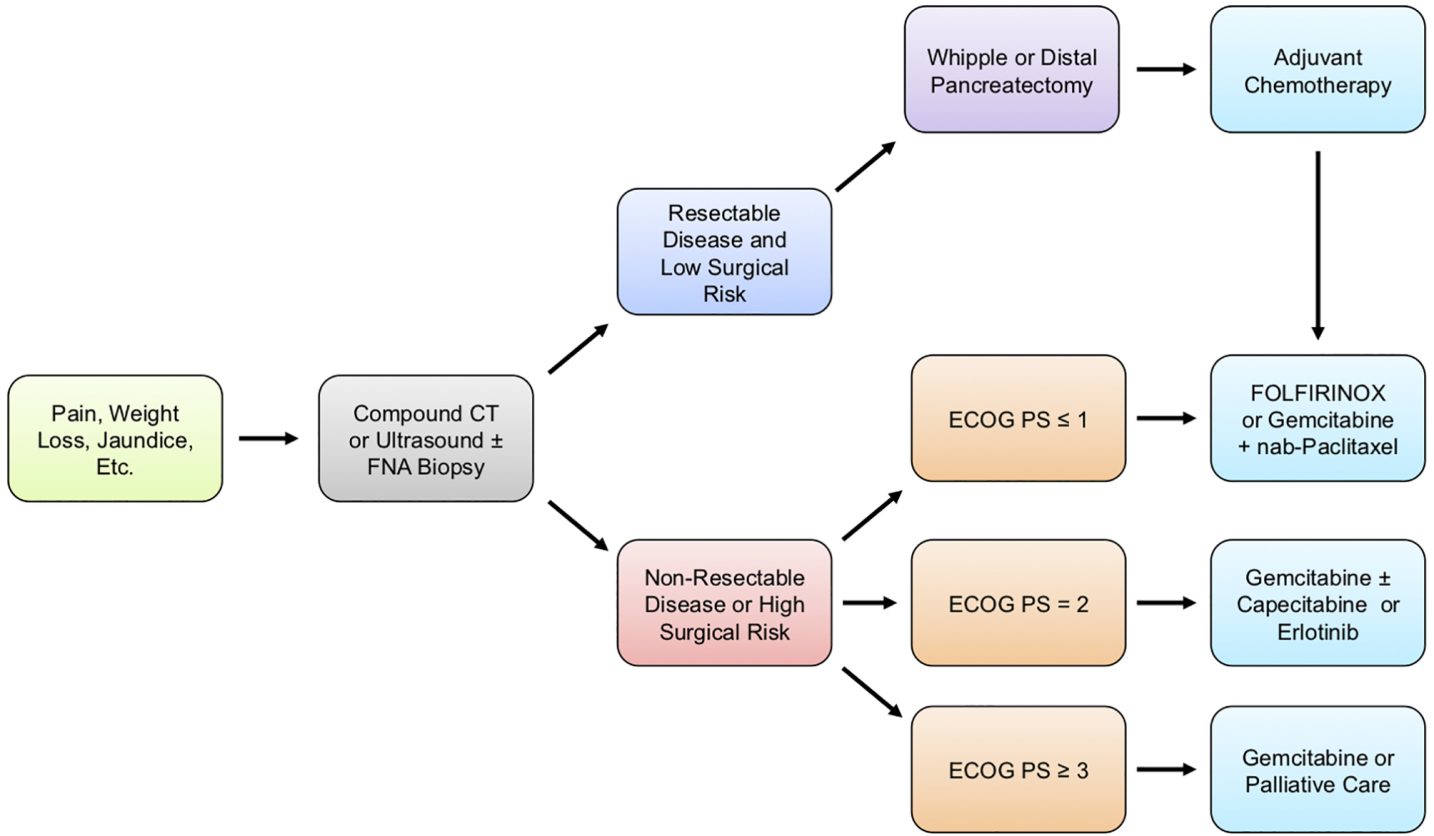
Generalized treatment guidelines for PDAC patients. Pancreatic ductal adenocarcinoma (PDAC) typically presents with vague clinical symptoms, including poorly localized pain, jaundice, or unintended weight loss. When PDAC is suspected, patients are typically diagnosed through computed tomography (CT) scan of the chest, abdomen, and pelvis to assess the extent of disease or ultrasound with or without a fine-needle aspiration (FNA) biopsy. Following confirmatory diagnosis, the patient’s surgical candidacy is determined based on a combination of imaging studies, Eastern Cooperative Oncology Group performance status (ECOG PS), symptom burden, surgical risk, and comorbidity profile. For operable disease, the type of surgery is determined based on the anatomical location of the tumor, as well as several additional factors described in this review article, with most patients receiving either a Whipple procedure or distal pancreatectomy. Regardless of whether a patient is treated with surgery, the current guidelines recommend chemotherapy, and the precise regimen given is based mostly on ECOG PS and comorbidity profile.

The clinical management of patients with pancreatic cancer varies depending on several factors, ranging from overall health and wellness to the wishes of the patient and family (**Figure 1**). During the initial patient assessment, a physician generally orders at minimum a computed tomography (CT) scan of the chest, abdomen, and pelvis in order to assess the extent of the disease. Before administering therapy, further steps are taken to determine patient performance status (PS), symptom burden, and comorbidity profile. Based on this information, as well as a discussion with the patient and their family, healthcare providers work to determine the overall goals of care and formulate a comprehensive treatment plan (12).

For metastatic disease, first-line treatment varies and is influenced largely by the Eastern Cooperative Oncology Group performance status (ECOG PS). For those seeking care with an ECOG PS of either 0 (Fully active, able to carry on all pre-disease performance without restriction) or 1 (Restricted in physically strenuous activity but ambulatory and able to carry out work of a light or sedentary nature, e.g., light housework, office work) (13), patients are typically offered either FOLFIRINOX or Gemcitabine plus Nab-Paclitaxel. For patients seeking treatment with an ECOG PS of 2 (Ambulatory and capable of all self-care but unable to carry out any work activities; up and about more than 50% of waking hours) or comorbidity profile that prevents the use of a more aggressive regimen, Gemcitabine is recommended in monotherapy, though agents such as Capecitabine can be offered in combination (12). For more severely disabled patients with an ECOG PS of 3 (Capable of only limited self-care; confined to a bed or chair more than 50% of waking hours) or 4 (Completely disabled; cannot carry on any self-care; totally confined to a bed or chair), or those with severe comorbidity, therapy is only offered on a case-by-case basis.

Second-line therapies are more varied and depend on additional factors, including patient preference and overall wellness. For patients who failed on first-line FOLFIRINOX and have an ECOG PS of <1 with the appropriate comorbidity profile, Gemcitabine plus Nab-Paclitaxel can be offered. Gemcitabine can also be offered alone as second-line therapy in patients with an ECOG PS of 2 or those with a substantial comorbidity profile that prevents the use of more aggressive regimens (12).

When indicated, Gemcitabine is administered *via* intravenous (IV) infusion at 1000 mg/m^2^ in four-week cycles, consisting of three once-weekly therapy followed by a break in the fourth week. While the number of cycles can vary, generally, postoperative patients undergo six cycles. Though the efficacy of Gemcitabine is significantly improved when used in combination with Nab-Paclitaxel, increasing 2-year-survival from 4% to 9%, this approach has significantly higher toxicities that must be considered. Patients treated with Gemcitabine and Nab-Paclitaxel had an increased rate of Grade 3 neutropenia (38% *vs.* 27%), febrile neutropenia (3% *vs.* 1%), fatigue (17% *vs.* 7%), and neuropathy (17% *vs.* 1%) (10). While these side effects are largely reversible, they often present a significant clinical challenge, namely a high risk of infection and reduced quality of life.

The combination of 5-FU/Leucovorin and nanoliposomal (Nal) Irinotecan has been approved by the FDA for patients who have been previously treated with Gemcitabine-based chemotherapy. This is based on recent clinical evidence showing that, in the second line, 5-FU/Leucovorin and Nal-Irinotecan offer a significant benefit to previously treated PDAC patients, extending median overall survival from 6.1 months compared to 4.2 months using 5-FU/Leucovorin (14). Importantly, this combination was well tolerated, and this study reported no new safety concerns, affirmed by a subsequent study in elderly patients (14, 15).

For patients with *BRCA1/2* or *PALB2*-mutated PDAC, front line therapy varies significantly. While only 5-9% of PDAC patients harbor such mutations, these patients appear highly sensitive to the combination of Gemcitabine and Cisplatin. This approach led to encouraging 2 and 3-year survival rates of 31% and 18%, respectively. As PARP inhibition can cause synthetic lethality in tumors with loss of high-fidelity double-strand break homologous recombination, the authors also explored the addition of the PARP inhibitor Veliparib, though this failed to further improve clinical outcomes. Though effective, the combination of Gemcitabine and Cisplatin was associated with a relatively high rate of Grade 3 to 4 hematologic toxicities, with 48% experiencing neutropenia, 55% thrombocytopenia, and 52% anemia (16). Also for BRCA-mutated patients, the PARP inhibitor Olaparib has shown significant efficacy as maintenance therapy, specifically for patients who had not progressed during first-line platinum-based chemotherapy (17). This approach extended median progression survival to 7.4 months compared to 3.8 months on placebo, with no difference in health-related quality of life (17).

Finally, patients deficient in DNA mismatch repair (dMMR) with high microsatellite instability (MSI-H) can respond to the immune checkpoint inhibitor Pembrolizumab. The KEYNOTE-158 trial reported that in dMMR/MSI-H PDAC patients, single agent Pembrolizumab had an overall response rate of 18.2%, with a median overall survival of 4 months, median progression-free survival of 2.1 months, and a median duration of response of 13.4 months (18). However, it is important to note that only ~1% of PDAC patients are dMMR/MSI-H (19), and that immunotherapy is not widely used in PDAC treatment at this time (20).

Additional precision medicine approaches are also beginning to show promise for PDAC, particularly for patients with *NTRK1–3* or *ROS1* gene fusions (21). These patients have shown increased sensitivity to selective tropomyosin receptor kinase (TRK) and ROS1 inhibitors larotrectinib and entrectinib (22). For example, the STARTRK-2 trial included two PDAC patients with a *TPR-NTRK* gene fusion, and one with an *SCL4-ROS1* gene fusion. These three patients derived substantial clinical benefit from entrectinib, showing either partial radiographic responses or stable disease (23). Similarly, the ongoing NAVIGATE trial evaluating larotrectinib in *NTRK* fusion-positive tumors also included one PDAC patient who showed a partial response by Response Evaluation Criteria in Solid Tumors (RECIST) criteria. Finally, select small molecule KRAS inhibitors are also emerging, particularly for tumors harboring a *KRAS^G12C^* mutation (24, 25). However, it is important to note that *KRAS^G12C^* mutations are exceptionally rare in PDAC, representing only 1% of all KRAS mutations (21, 26). An alternative strategy to target more common *KRAS* mutations is also under clinical investigation. This approach uses exosomes loaded with small interfering RNAs against *KRAS^G12D^*, the most common KRAS mutation in PDAC, and has shown encouraging preclinical efficacy (27).

## Surgery

Surgery offers a significant survival benefit to eligible patients, particularly when combined with adjuvant chemotherapy (28). In contrast to the overall 5-year survival rate of ~10% for all patients, patients undergoing resection for stage I PDAC have an overall 5-year survival of 38.2% compared to 2.9% for patients who did not (29, 30). Globally, rates of resection for Stage I/II disease vary, ranging from 35% to 69% (31). These variances appear to be due to several added factors, including performance status, tumor size and location (31), and socioeconomic factors such as race (32). Next, we provide a high-level overview of the surgical management of operable PDAC and emerging barriers that may hinder the utility of surgery in the clinical management of PDAC.

### Surgical Candidacy

Surgery with perioperative chemotherapy remains the only treatment option for achieving long-term survival for patients with PDAC. Unfortunately, only 15-20% of patients are candidates for upfront surgical resection, while 30-40% have unresectable/locally advanced disease and 50-60% have distant metastases at presentation (33). Even for those who do undergo surgery, 5-year survival remains dismal at 24% (29). Given these observations, at the time of diagnosis, patient disease state is classified as resectable, borderline resectable, locally advanced, or metastatic.

**Figure 2:**
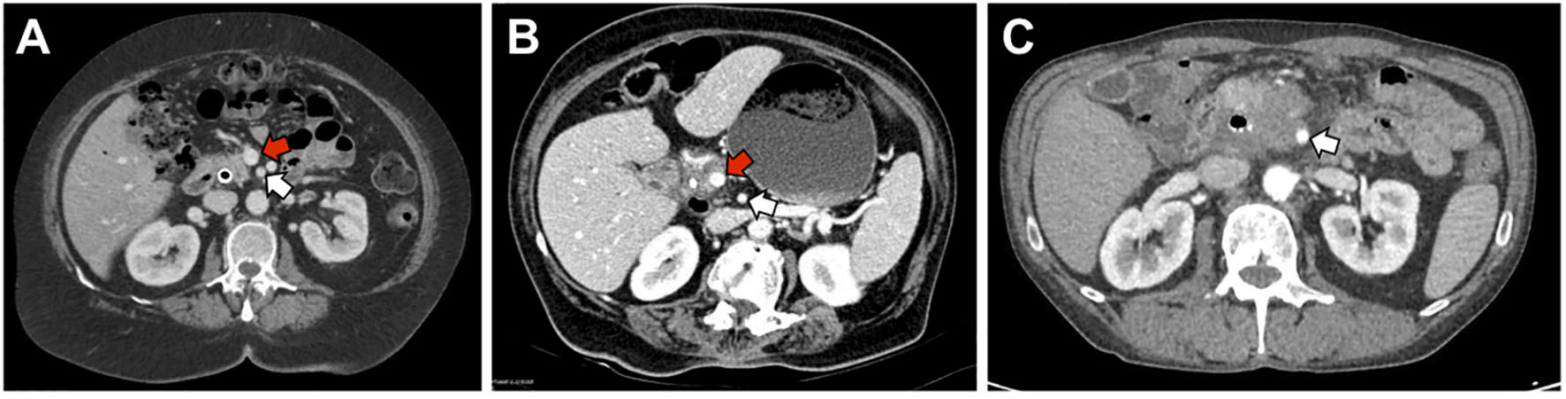
Representative images of resectable, borderline-resectable, and non-resectable PDAC. **(A)** Representative image of a patient with resectable disease as defined by NCCN guidelines. There is no tumor contact with the superior mesenteric artery (SMA, white arrow) and <180° of tumor contact with the superior mesenteric vein (SMV, red arrow). **(B)** Patient with borderline resectable disease due to >180° of tumor contact with the SMV but no involvement of the SMA. **(C)** Patient with locally advanced disease due to >180° of tumor contact with the SMA.

Patients are considered resectable if there is no evidence of distance metastasis, no arterial tumor contact with the celiac axis (CA), superior mesenteric artery (SMA), or common hepatic artery (CHA), and no tumor contact greater than 180° with the superior mesenteric vein (SMV) or portal vein (PV) (**Figure 2**). Additionally, there should be no vein contour irregularity for patients with less than 180° contact with the SMV or PV (34). For these patients, the standard-of-care has been upfront surgery followed by adjuvant chemotherapy. Even for those who undergo surgery for resectable disease, recurrence rates are 76.7% after two years (35).

More recently, there is an emerging hypothesis that response to neoadjuvant chemotherapy can be used as a test of underlying tumor biology to identify patients who would benefit from surgical resection. A recent meta-analysis of eight cohort studies and 3 randomized controlled trials (RCT) of neoadjuvant therapy in resectable PDAC found an increased R0 resection rate but no survival benefit (36). A sub-analysis of patients receiving neoadjuvant gemcitabine found a survival benefit compared to upfront resection (HR 0.75, 95%CI 0.73-1.03). A more recent randomized phase III trial in the Netherlands found a lack of survival benefit in patients receiving neoadjuvant gemcitabine compared to upfront resection but improvement in secondary outcomes such as R0 resection (71% *vs.* 40%) and disease-free survival (37). Other clinical trials are ongoing and will help determine the appropriateness of neoadjuvant therapy for resectable PDAC (38). Additionally, neoadjuvant therapy should also be considered for patients with high-risk features such as high levels of serum CA 19-9, a large primary tumor, large regional lymph nodes, excessive weight loss, or extreme pain (34, 39, 40).

There is a category of patients who are resectable by imaging criteria but considered to be physiologically unresectable based on advanced age, frailty, comorbidities, and performance status. As might be expected, patients over the age of 80 have a significantly increased risk of mortality after surgery, although surgery continues to provide a survival benefit in these patients (41). Several studies have suggested that frail patients and those with poor performance status have worse morbidity, mortality, and survival after surgery for PDAC (42–44). However, there should be no strict age limit for surgical resection, and appropriateness for resection is best determined by evaluating patient factors, life expectancy, and properly counseling the patient.

Patients with borderline resectable disease have solid tumor contact with CHA or SMA < 180°, solid tumor contact with SMV or PV >180°, solid tumor contact with SMV or PV <180° with contour irregularity or vein thrombosis that is suitable for vessel resection, or solid tumor contact with the IVC (34). These patients typically require upfront tissue diagnosis and proceed to neoadjuvant chemotherapy after the staging is complete. Patients who respond to therapy may go on to surgical resection while those with disease progression continue with non-operative management of their disease. The optimal neoadjuvant regimen is unknown but typically involves FOLFIRINOX or Gemcitabine-based regimens. After completion of neoadjuvant chemotherapy, resection may be considered if there is no evidence of metastatic disease, no progression of disease, no more than mild increase in perivascular soft tissue, and stable or decreasing CA 19-9 (34). Neoadjuvant therapy in this population results in an approximate rate of resection of 60-70% (45–47). Neoadjuvant therapy results in an improved R0 resection rate, reduced nodal disease, and improved overall survival in this patient population (45, 48). Additionally, it may reduce the rate of futile surgery in those who progress while on chemotherapy.

Patients with locally advanced disease have >180° contact with the SMA or CA, aortic involvement, or unreconstructable involvement of the SMV/PV (34). For those with poor performance status, palliative and supportive care are best. For those with good performance status, NCCN guidelines recommend enrollment in a clinical trial of neoadjuvant chemoradiation (34). Patients should be considered for surgery after neoadjuvant therapy if there is >50% decrease in CA 19-9 and improvement in patient factors such as pain, early satiety, nutritional status, and performance status (34). The resection rate in this population is much lower than for those with the borderline resectable disease at about 28%, with a reported range of 0-40% (39, 49, 50). Patients with distant metastasis involving the liver, peritoneum, or omentum are generally considered unresectable. While there are centers exploring metastectomy for patients with oligometastatic PDAC in select patients, these data are limited to cohort studies (51, 52).

### Disparities and the Underutilization of Surgery

Determining candidacy for surgical resection is best done using a multi-disciplinary approach at an experienced center, as variability in treatment and surgical utilization contributes to poor outcomes in PDAC. For instance, a 2007 study of the National Cancer Database found that a staggering 71.4% of patients with Stage I disease did not undergo surgery. Patients above the age of 65, African American patients, and patients on Medicare or Medicaid were less likely to undergo surgery (29). Other studies have confirmed that socioeconomic variables such as income, education, insurance, and treatment facility are associated with failure to receive standard treatment and worse clinical outcomes (53–55).

Patients receive expected treatment more often and have improved outcomes with treatment at medical centers with surgeons who perform a high volume of pancreatic surgeries (56–58). This raises the issue of geographic disparity, as patients who travel farther to high volume centers have improved survival than those who stay closer to home at lower volume centers, despite having more advanced disease (59). Health disparities also exist in the referral of patients to these high-volume centers (57, 60, 61). For example, patients from socioeconomically disadvantaged backgrounds and minority patients are less likely to be referred to high-volume centers, thereby contributing to their poorer outcomes (57, 60). Accordingly, centralization of PDAC care to high volume centers may address this disparity and improve outcomes, and efforts are currently ongoing to improve access to care at high volume hospitals (62).

### Preoperative Biliary Drainage

Obstructive jaundice is a frequent complication for patients with pancreatic head cancers and can be relieved by preoperative biliary drainage. However, for patients going to surgery, a multicenter RCT demonstrated an increased risk of serious complications for those who underwent preoperative biliary drainage (74%) compared to those who went directly to surgery (39%) within 1 week of diagnosis (63), a finding corroborated by multiple meta-analyses (64, 65). As mentioned, the use of neoadjuvant chemotherapy is rising for patients with resectable PDAC. However, these patients often require biliary drainage for symptom palliation during neoadjuvant treatment. Therefore, the number of patients with biliary stents in place at the time of surgery is likely to increase, and this topic warrants continued exploration.

### Perioperative Nutrition Management

A significant proportion of PDAC patients will develop cancer-associated cachexia and malnutrition (66), conditions associated with poor clinical outcomes (42, 67). Despite this observation, there is a lack of data evaluating preoperative nutritional interventions to improve postoperative outcomes in patients with PDAC, though the role of postoperative nutritional interventions is more established. Interestingly, there is little data to support routine enteral or parenteral nutrition in patients following pancreatic resection. Somewhat related, a multicenter European RCT compared early enteral feeding to parenteral feeding in patients after pancreaticoduodenectomy (PD) (68). Early enteral feeding was associated with increased frequency and severity of postoperative pancreatic fistula (POPF), while parenteral feeding patients had earlier recovery of oral feeding. Unfortunately, this study did not include an oral feeding group, which is supported by the Enhanced Recovery After Surgery Society recommendations (69). Several studies have reported successful resumption of oral diet early in the post-operative course without the need for enteral or parenteral nutrition (70–72). Hence, though a randomized control trial is needed, early oral feeding appears safe and does not worsen the duration or grade of POPF (72).

### Staging Laparoscopy

The decision to perform staging laparoscopy is made on a case-by-case basis. Patients at the highest risk of unresectable disease are most likely to benefit from staging laparoscopy, which detects occult metastatic disease, such as small liver or peritoneal metastasis, not appreciated on preoperative imaging. Staging laparoscopy identifies occult metastatic disease in 8-26% of patients, saving a need for exploratory laparotomy (73, 74). As risk factors for the occult metastatic disease include abdominal pain, tumor size > 30 mm, indeterminate liver lesions, and CA 19-9 level > 192 U/ml (40, 73), the presence of such risk factors indicates a need for staging laparoscopy. As neoadjuvant approaches are increasingly used for patients with borderline resectable and locally advanced disease, patients at higher risk for occult metastatic disease are more likely to undergo surgery. The use of staging laparoscopy in this population will be important, however, with some suggesting staging laparoscopy before neoadjuvant therapy as a mechanism to diagnose occult metastatic disease earlier in the disease course (75).

The addition of laparoscopic ultrasound to staging laparoscopy has been proposed as a possible adjunct to staging laparoscopy. It appears to provide additional prognostic information in a minority of patients and should not be routinely performed (76). Novel imaging techniques, such as near-infrared fluorescence imaging, to detect occult metastasis or unresectable disease are now being developed (77, 78), though further evidence is required before the implementation of these techniques.

### Palliative Surgery

For patients who are found to have inoperable disease on staging laparoscopy or laparotomy, the surgeon must decide whether to perform surgical palliation. Classically, a hepaticojejunostomy and gastrojejunostomy were performed for the prevention of biliary and gastric outlet obstruction. As advanced endoscopic techniques have improved, the need for these operations has come into question. For patients with obstructive jaundice, placement of self-expanding metals stents (SEMS) has become the gold standard due to its lower morbidity, but there is a higher rate of recurrent obstruction and need for repeat intervention than surgical bypass (79, 80). For the patient with obstructive jaundice and found to be inoperable at the time of surgery, a surgical biliary bypass is recommended (34). The role of a prophylactic biliary bypass is unclear and should be at the discretion of the surgeon and patient.

For patients with malignant gastric outlet obstruction (GOO) and a life expectancy >2 months, there is better long-term relief of symptoms with gastrojejunostomy (GJJ) than endoscopic stenting (81). Again, the role of prophylactic GJJ is less clear. Early literature suggested an improvement in future GOO symptoms with GJJ (82, 83). As advanced endoscopic techniques and stents have improved, a “wait-and-see” strategy has been developed for both obstructive jaundice and GOO, wherein patients receive SEMS at the time of biliary obstruction and treats only symptomatic GOO (84). The wait-and-see approach was associated with lower morbidity and hospital length of stay and a similar need for reintervention compared to prophylactic surgery of both conditions. Prophylactic GJJ did not prevent future GOO in this study. Patient factors and patient counseling play a vital role in surgical decision-making for these patients.

### Minimally Invasive Surgery

Recent advances in minimally invasive surgery have prompted the use of laparoscopic and robotic techniques for pancreatic resections. Compared to many other surgical procedures, any benefit of minimally invasive techniques in pancreatic head resections is subtle. There is a significant learning curve associated with the minimally invasive techniques. For PD, the learning curve is between 40-80 cases (85, 86). Even at high volume centers, this is a time-consuming and challenging endeavor, and outcomes early in the learning curve are worsened (87). In experienced hands, minimally invasive PD appears to have lower EBL, shorter length of stay, similar morbidity/mortality, and similar oncologic outcomes (88–91).

A recent RCT compared minimally invasive techniques to open distal pancreatectomy (OPD). The LEOPARD trial found shorter length of stay, less delayed gastric emptying (DGE), better quality of life scores, and similar morbidity in patients undergoing minimally invasive distal pancreatectomy (MIPD) (92). The DIPLOMA study was a score-matched study of 1,212 patients undergoing MIPD or OPD. Similarly, they found a shorter length of stay, comparable morbidity/mortality, and similar overall survival between the two groups (93). The majority of patients undergoing MIPD are undergoing laparoscopic surgery. There is insufficient evidence to appropriately evaluate robotic *vs.* laparoscopic distal pancreatectomy at this time.

### Vascular Resection and Reconstruction

Patients with PDAC and PV or SMV involvement were previously felt to be unresectable. Recent literature has called this into question. Although patients undergoing PD with PV/SMV resection likely have increased morbidity and mortality (94, 95), two meta-analyses found comparable survival to those that did not require resection (96, 97). At high-volume pancreatic surgery centers, venous resection and reconstruction appears safe and may prolong survival. As these patients are considered locally advanced, patients should undergo neoadjuvant therapy before surgical resection (34).

As patients requiring venous resection/reconstruction are now considered resectable, some have advocated for an “artery-first” approach to PD (98). This technique involves up-front careful dissection of involved arterial planes to determine resectability. A meta-analysis of this approach compared to conventional PD found increased R0 resection rates and survival (99). This study was limited primarily to retrospective cohort studies or case-control studies. A more recent multicenter RCT was found no difference in R0 resection rate, morbidity, or mortality. Further evidence is required to support an artery-first approach.

Arterial resection during PD for PDAC remains highly controversial but is performed for select patients with limited involvement of hepatic arteries at some centers (100–102). Patients requiring arterial resection appear to have poor short and long-term survival (101). Further evidence is needed to determine any survival benefit in patients requiring arterial resection compared to no surgery. For patients undergoing distal pancreatectomy (DP), resection of the celiac axis can be performed when there is no tumor involvement of the celiac artery origin or the confluence of the gastroduodenal artery (GDA) and CHA. A meta-analysis revealed that DP with resection of the celiac axis is associated with similar morbidity/mortality and survival compared to DP (103). This strategy should be reserved for select patients performed at high volume centers.

### Lymphadenectomy

The extent of lymphadenectomy for PD has been debated. Standard lymphadenectomy includes lymph node stations 5, 6, 8a, 12b1, 12b2, 12c, 13a, 13b, 14a, 14b, 17a, and 17b (104). Multiple RCTs and meta-analyses have failed to show any survival benefit for patients undergoing extended lymphadenectomy compared to standard lymphadenectomy and may have associated morbidity (105–109). Morbidity included an increase in POPF, DGE, length of stay, and a decrease in quality of life scores (105, 107, 108). Resultantly, current evidence does not support performing an extended lymphadenectomy.

### Total Pancreatectomy

Total pancreatectomy is occasionally necessary for more advanced cancers or large central tumors to obtain negative margins. There are obvious clinical implications of removing the entire pancreas, including brittle diabetes and pancreatic exocrine insufficiency. Patients who undergo total pancreatectomy compared to partial pancreatectomy appear to have similar mortality and long-term survival with an increased rate of margin-negative resection (110–112). However, the quality of life after total pancreatectomy is slightly lower than the general population or those undergoing partial pancreatectomy (113, 114). This is driven by diabetes-associated factors as well as diarrhea. Given the mortality and survival in patients undergoing total pancreatectomy, the operation is only warranted when absolutely necessary to obtain negative margins.

### Adjuvant Therapy

The ability to complete adjuvant chemotherapy becomes an important consideration after surgery. Completion of adjuvant therapy after R0 resection is associated with improved survival (115, 116). Unfortunately, many patients are unable to complete adjuvant chemotherapy. A study of the SEER database determined that only 7% of Medicare patients completed adjuvant chemotherapy after upfront resection (115). A retrospective study of 309 patients from a single center found 81% initiated adjuvant therapy while 65% completed the recommend course (117). A randomized trial comparing 6 cycles of gemcitabine to observation found that 61% of patients randomized to gemcitabine received all 6 cycles (116). Many patients have delayed postoperative courses due to the morbidity of pancreatic resections. Interestingly, the time to initiation of adjuvant therapy does not affect survival (118–121). Patients with delays in receiving adjuvant treatment should still be considered candidates for therapy.

Adjuvant chemotherapy can vary significantly. Several recent studies have explored the comparative efficacy of standard of care approaches for PDAC. For instance, a recent phase III trial of 493 patients explored the comparative efficacy of a modified FOLFIRINOX regimen to Gemcitabine monotherapy in patients with resected PDAC. After a median follow-up of 33.6 months, modified-FOLFIRINOX led to a median disease-free survival of 21.6 months compared to 12.8 months in with Gemcitabine. However, modified-FOLFIRINOX was associated with a higher incidence of adverse effects, with 75.9% of patients in the modified-FOLFIRINOX group experiencing a Grade 3/4 toxicity compared to 52.9% in the Gemcitabine group (11). The phase III ESPAC-4 trial also explored the combination of adjuvant Gemcitabine plus Capecitabine with Gemcitabine monotherapy in 732 patients with recently resected PDAC. The authors reported a median overall survival of 28 months in patients treated with Gemcitabine plus Capecitabine, and 25.5 months in the Gemcitabine group. Grade 3/4 adverse events were observed in 63% of the Gemcitabine plus Capecitabine arm, and 53.5% of the Gemcitabine arm (122). Hence, additional study is warranted to better determine the optimal treatment in the adjuvant setting.

## Pancreatic Cancer Chemotherapy

As mentioned, nearly all intent-to-treat patients will receive multi-agent chemotherapy. This has long been the backbone of pancreatic cancer management. While a variety of small molecule and immune checkpoint inhibitors have shown tremendous efficacy in other cancer types, such approaches generally lack supportive evidence in PDAC and most patients are managed through chemotherapy alone. For all the advances in our understanding of PDAC pathobiology, the chemotherapy agents used in PDAC patients are several decades old, with the most recent breakthroughs consisting of new combinations of existing medications. Here, we describe the discovery and mechanism of action for the most frequently used PDAC medications, as well as any emerging mechanisms of resistance in hopes that these may be used to identify more novel, targeted approaches to improve clinical response rates.

## Gemcitabine

Gemcitabine is among the most widely used medications in the management of PDAC. It was first developed in the 1980s by Eli Lilly as an antiviral drug. After showing potent anti-neoplastic effects *in vitro*, Gemcitabine soon entered various clinical trials in the 1990s. Showing clear efficacy in pancreatic cancer patients, Gemcitabine was first approved for use in PDAC in the United Kingdom in 1995 and then by the FDA in 1996. In 1998, Gemcitabine was approved in combination with Cisplatin for first-line treatment of patients with inoperable non-small cell lung cancer. In 2004, Gemcitabine was next approved for the treatment of metastatic breast cancer following failure of prior anthracycline-containing adjuvant chemotherapy and in 2010, approved in combination with carboplatin for use in select ovarian cancer patients. While Gemcitabine is indeed approved for these and other malignancies, it is most commonly used in combination with Nab-Paclitaxel in advanced PDAC.

**Figure 3:**
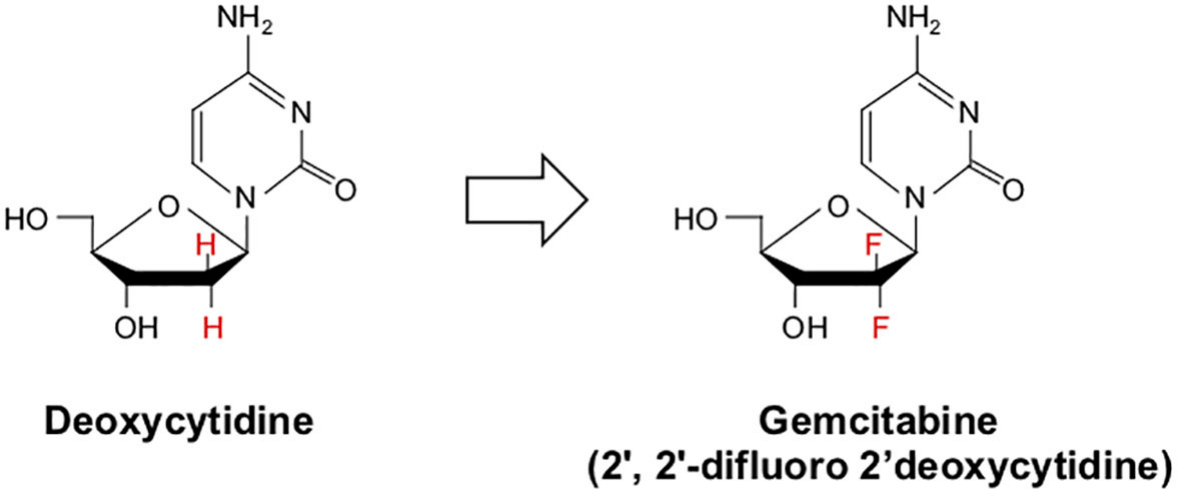
Chemical structure of deoxycytidine and its halogenated chemical mimic Gemcitabine. Gemcitabine (2’, 2’-difluoro 2’deoxycytidine or dFdC) is a nucleoside analog identical in structure to deoxycytidine apart from two fluoride molecules at the 2’-position.

Gemcitabine (2’, 2’-difluoro 2’deoxycytidine or dFdC) is a nucleoside analog that is identical in structure to deoxycytidine apart from two fluoride molecules at the 2’ position (**Figure 3**). Gemcitabine enters cells *via* several nucleotide transporters, including SLC29A1, SLC28A1, and SLC28A3. However, most appear to be taken into the cell *via* Human Equilibrative Nucleoside Transporter 1 (hENT1). Once in the cytoplasm, Gemcitabine is phosphorylated by Deoxycytidine Kinase (dCK) to form dFdC monophosphate (dFdCMP), the substrate for two distinct reactions. This initial phosphorylation by dCK is the rate-limiting step in Gemcitabine metabolism. dFdCMP is then either deaminated by Deoxycytidylate Deaminase and converted to dFdUMP, or phosphorylated by Nucleoside Monophosphate Kinase to become dFdC diphosphate (dFdCDP). dFdUMP is a potent inhibitor of the enzyme Thymidylate Synthase. Classically, Thymidylate Synthase catalyzes the conversion of deoxyuridine monophosphate (dUMP) to deoxythymidine monophosphate (dTMP). dFdUMP competitively inhibits Thymidylate Synthase, thereby causing an imbalance between dUMP and dTMP, impeding DNA synthesis and promoting DNA damage, culminating in programmed cell death (123).

**Figure 4:**
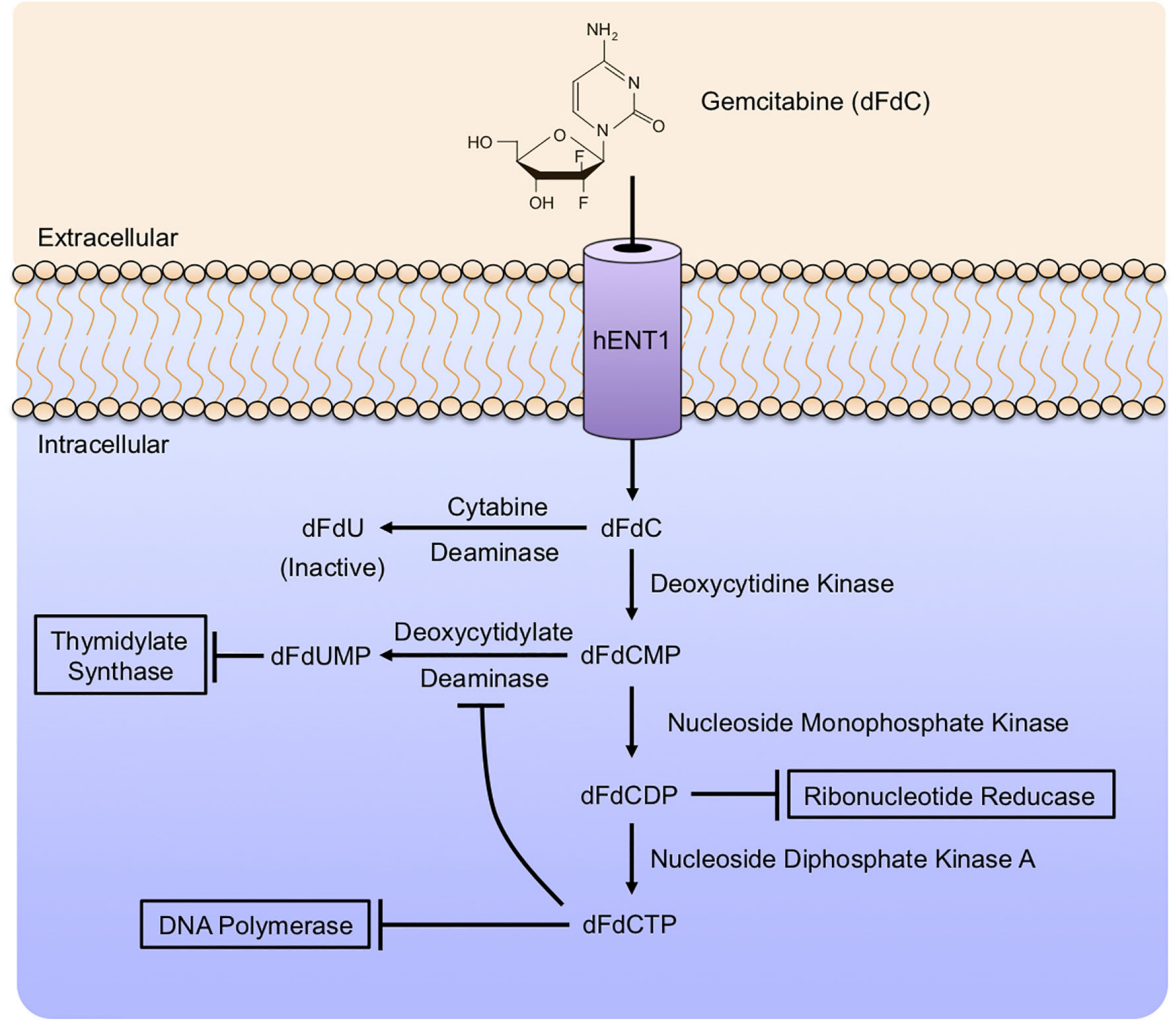
Gemcitabine mechanism of action. Gemcitabine enters cells *via* several nucleotide transporters, primarily Human Equilibrative Nucleoside Transporter 1 (hENT1). In the cytoplasm, Gemcitabine is modified extensively by a series of enzymatic reactions. Gemcitabine is phosphorylated by Deoxycytidine Kinase (dCK) to form dFdC monophosphate (dFdCMP), the rate-limiting step in Gemcitabine metabolism. Subsequently, dFdCMP can be deaminated by Deoxycytidylate Deaminase to form dFdUMP, a potent inhibitor of Thymidylate Synthase. Alternatively, dFdCMP can be phosphorylated by Nucleoside Monophosphate Kinase to become dFdC diphosphate (dFdCDP), inhibiting Ribonucleotide Reductase. dFdCDP can be further phosphorylated by Nucleoside Monophosphate Kinase A to form dFdCTP, which inhibits DNA polymerases. As an alternative to these activating modifications, Gemcitabine can be deaminated by Deoxycytidylate Deaminase to form dFdU, an inactive metabolite with no known anti-neoplastic effects.

Alternatively, dFdCDP impairs the function of Ribonucleotide Reductase (RNR). RNR is required for DNA synthesis, reducing the 2’-hydroxyl group of the ribose ring of ribonucleoside 5’-diphosphates (ADP, CDP, GDP, and UDP) for *de novo* synthesis of deoxyribonucleotides. This is critical for DNA synthesis and cell proliferation, and RNR is a predictor of poor outcomes in PDAC patients (124). Additionally, dFdCDP can be further phosphorylated by Nucleoside Monophosphate Kinase to become dFdC triphosphate (dFdCTP). dFdCTP is considered the most important Gemcitabine metabolite and acts by incorporation into genomic DNA. This results in the inhibition of DNA polymerases, preventing DNA synthesis and repair (125). These events are summarized in **Figure 4**. Gemcitabine is primarily detoxified in the liver. Upon entry into hepatocytes, dFdC is deaminated by Cytidine Deaminase (CDA) to form the inactive 2’-difluoro 2’deoxyuracil (dFdU) metabolite. Following its conversion, dFdU is then eliminated by both renal and biliary excretion (126).

### Gemcitabine Resistance

Gemcitabine has been a standard-of-care agent in the management of advanced PDAC for decades. While Gemcitabine offers a survival benefit both as a monotherapy and in combination with other medications, these responses are seldom complete, and nearly all tumors display or will develop some degree of Gemcitabine resistance, with only 4% of patients surviving two years on Gemcitabine alone (10). Though Gemcitabine resistance is well documented clinically, the cellular and molecular mechanisms that underlie Gemcitabine resistant phenotypes remain unclear. Several studies have attempted to predict for Gemcitabine responses, though there are no such biomarkers clinically used at this time.

As discussed, the main method of Gemcitabine uptake is *via* the hENT1 transport protein. Accordingly, hENT1 expression has been suggested to predict for response to Gemcitabine therapy in the adjuvant setting. A retrospective meta-analysis compiled 10 studies and determined that hENT1 positively associated with improved overall survival with a hazard ratio of 0.52 in patients treated with Gemcitabine (127). The majority of trials evaluated in meta-analysis used the 10D7G2 mouse monoclonal antibody to determine hENT1 status by immunohistochemistry. Unfortunately, this antibody has no commercially available source for larger trials. To this end, a similar SP120 rabbit monoclonal anti-hENT1 antibody was generated to prospectively predict Gemcitabine responsiveness in metastatic disease. However, positive staining with SP120 showed no correlation between hENT1 expression and response to Gemcitabine. Additionally, the 11337-1-AP antibody also had a low success rate, suggesting that the predictive value of hENT1 is highly antibody-dependent (128).

While incomplete Gemcitabine uptake *via* loss of hENT1 may partially explain Gemcitabine resistance, emerging evidence suggests that there are several other factors that may contribute to drug responses. For instance, while the Gemcitabine inactivating enzyme CDA is mainly expressed in the liver, it is also expressed in various tissues, including the bone marrow, prostate, pancreas, and spleen (129). Genomic sequencing further suggests that pharmacogenomic variation in CDA may contribute to the therapeutic efficacy of Gemcitabine, though this requires further study and is not currently used to predict Gemcitabine responses in the clinic (129). Additionally, in some patients, Gemcitabine resistance appears to be mediated by efflux pumps, namely the ABC transporter family proteins ABCB1/MDR1, ABCC1/MRP1, and ABCG2/BCRP (130).

Other genomic alterations affecting Gemcitabine transport and metabolism have also been identified, provided further insight into Gemcitabine resistant phenotypes. For instance, Cytosolic 5’-nucleotidase 1A (NT5C1A) is a phosphatase that targets non-cyclic nucleoside monophosphates to produce nucleosides and inorganic phosphates. NT5C1A is robustly expressed in tumor cells of resected PDAC patients. Interestingly, NT5C1A promotes Gemcitabine resistance by decreasing the amount of intracellular dFdCTP and limits therapeutic responses to Gemcitabine in mice (131). Additionally, expression of the tumor suppressor Hepatocyte nuclear factor 1α (HNF1A) seems to associate with Gemcitabine sensitivity in PDAC cells, regulating the expression of ABCB1 and ABCC1. The authors further demonstrated that HNF1A regulates *ABCB1* gene expression by binding its promoter region and suppressing its transcription, suggesting that HNF1A warrants exploration as a potential biomarker for Gemcitabine responses in the clinic.

Beyond alterations to nucleoside metabolism enzymes and drug transport, myriad signaling events have also been linked to Gemcitabine-resistant phenotypes. These include several cell survival pathways, namely AKT (132, 133), ERK/MAPK (134–136), HIF1α (137), GLI and SOX2 (138), NFκB (132, 139, 140), Sonic Hedgehog (141), and WNT (142, 143). Additionally, several recent preclinical studies have identified select microRNAs that may also contribute to Gemcitabine-resistance. For example, miR-21 is upregulated in Gemcitabine-refractory PDAC and is believed to contribute to drug-resistant phenotypes through a variety of mechanisms (144). Conversely, members of the miR-200 family are generally repressed in Gemcitabine-resistant cells and, when restored, promotes the reversion of post-EMT tumor cells to a more epithelial state (145, 146). Interestingly, inactivation of the Hippo tumor suppressor gene has been suggested to regulate cell-density-dependent miRNA suppression in cancer *via* de-repression of YAP (147). However, additional evidence suggests that YAP enhances the actions of Gemcitabine, largely by down-regulating multi-drug transporters. Similarly, cell lines with genetic ablation of Hippo signaling had increased sensitivity to Gemcitabine (148). These data suggest that the contributions of Hippo-mediated microRNA biogenesis to Gemcitabine resistance are complex and highly varied and warrant further study. Several additional mechanisms of resistance have also been suggested, including aberrations to the epigenome. For instance, one study determined that while Gemcitabine-resistant PDAC cell lines acquire an invasive phenotype and associated upregulation of CDA, these cell lines also displayed collateral hypersensitivity to histone deacetylase (HDAC) inhibitors and decreased expression of heterochromatin marks H4K20me3, H3K9me3, and H3K27me3 (149).

Recently, a group of investigators have conducted genome-wide RNA profiling of primary PDAC cell cultures and patient-derived xenografts, and related them to Gemcitabine sensitivity. Using this approach, the authors identified a unique gene expression signature associated with Gemcitabine sensitivity, which they designated GemPred. They then tested the GemPred RNA signature first in a monocentric cohort of 67 patients, then in a multicentric cohort of 368 patients. In both, GemPred+ patients who received Gemcitabine had significantly longer overall survival than those who were GemPred-. Additionally, GemPred stratification was not associated with a survival benefit in patients not receiving Gemcitabine. The authors therefore concluded that GemPred stratification predicts the benefit of adjuvant Gemcitabine in PDAC, and this approach warrants continued exploration in prospective studies (150).

Emerging evidence also suggests that the glycan biomarker sialylated tumor-related antigen (sTRA) may have utility in predicting responses to Gemcitabine, as well as several other chemotherapeutics used in PDAC. For instance, sTRA-expressing cell lines were associated with increased resistance to seven different chemotherapeutics used in PDAC, and patients with primary tumors were positive for a gene expression classifier for sTRA demonstrated no statistically significant benefit from adjuvant chemotherapy. Similarly, plasma levels of sTRA identified the PDACs that showed rapid relapse following neoadjuvant chemotherapy. Hence, pending further evaluation, sTRA may be a useful means of predicting for drug responses in PDAC patents (151).

While the above events have been well characterized, it has also been suggested that the tumor microenvironment (TME) may have many important roles in drug responses (152). PDAC is associated with a dense, reactive tumor stroma comprised of activated stellate cells, cancer-associated fibroblasts, and leukocytes (153, 154), and tumors from Gemcitabine-resistant patients appear to generally be enriched in stromal pathways (155). In addition to its role in producing a variety of growth factors and cytokines, such as hepatocyte growth factor (HGF) and transforming growth factor β (TGFβ) (156, 157), the PDAC stroma is known to impede drug delivery through several mechanisms, namely by exerting considerable mechanical force on the intratumoral vasculature and reducing patency (158). Both functions have been suggested as potential drug targets to enhance the effects of Gemcitabine.

For instance, TP53 is mutated in 75% of PDAC tumors and is implicated in a number of hallmark features of tumorigenesis (159). In mice, loss-of-function TP53 mutations were associated with hyper-activation of the JAK2/STAT3 signaling cascade, leading to extensive stromal remodeling and Gemcitabine-resistant phenotypes (160). Similarly, STAT3 activation associated with poor survival in human PDAC and inhibition of this pathway reduced the tumor stroma and sensitized tumor-bearing mice to Gemcitabine (160). Additionally, Stromal Cell-Derived Factor 1 (SDF-1 or CXCL12) and its receptor CXCR4 may also have a potential role in Gemcitabine resistance. Stromal-derived SDF-1 appears to bind CXCR4 on the surface of the tumor epithelium, thereby activating AKT, ERK, and IL6-mediated survival pathways (161). Several other studies have suggested a role for such paracrine signaling events in Gemcitabine resistance, namely as blocking stromal protein synthesis *via* mTOR/4E-BP1 inhibition led to a reduction in tumor IL6, as well as the loss of drug-resistant phenotypes (162).

Additional evidence suggests that the desmoplastic component of the tumor stroma may also contribute to Gemcitabine resistance. In *in vitro* systems, tumor cells grown in 3D collagen culture are able to override Gemcitabine-induced cell cycle arrest through MMP-14-induced expression of HMGA2 (163). However, while the biologic contribution of the stroma to Gemcitabine resistance is well established, the clinical utility of stromal depletion remains unclear. For example, catabolism of the tumor stroma induced by the Hedgehog inhibitor IPI-926 led to transient increases in intratumoral vascular density and improved the uptake of Gemcitabine (141). However, additional evidence suggests that tumors with genetic deletion of Hedgehog signals are highly aggressive, with poor differentiation and increased vascularity. These events suggest that the cancer stroma may restrain tumor growth, in part by impeding tumor angiogenesis (164). This is supported by clinical data, as the Sonic Hedgehog antagonist Vismodegib failed to improve either Gemcitabine response rates or survival in patients with metastatic PDAC (165).

While most research efforts have focused on pancreatic cancer-associated fibroblasts and stellate cells, emerging evidence appears to suggest that a variety of additional cell types within the tumor microenvironment may also contribute to Gemcitabine resistance. While PDAC is generally non-immunogenic, tumors are associated with robust macrophage infiltration. Tumor-educated macrophages appear to release several pyrimidine species, including deoxycytidine, which appear to inhibit the uptake and action of Gemcitabine in tumor cells. Similarly, genetic or pharmacologic depletion of macrophages sensitized tumor-bearing mice to Gemcitabine, further substantiating a potential role for the immune infiltrate in Gemcitabine resistance (166).

Interestingly, a recent study determined that select species of proteobacter expressing CDA internalize and metabolize Gemcitabine to its inactive form (167). These bacterial species were found in a significant fraction of tumor specimens, particularly those with intervention of the main pancreatic duct. As these are commensal duodenal flora, it is likely that the manipulation of the main pancreatic duct *via* the duodenum introduces CDA-expressing bacteria to the pancreas, potentially limiting the efficacy of Gemcitabine. Both *in vitro* and *in vivo* studies suggest that the addition of select antibiotics may augment the tumoricidal action of Gemcitabine in infected tumor cells, though this has yet to be explored in large scale, randomized testing (167).

### Strategies to Overcome Gemcitabine Resistance

For patients receiving Gemcitabine-based chemotherapy, the eventual development of drug-resistant phenotypes is a significant issue. While several of the mechanisms described above have been proposed as potential targets for therapy, far fewer have been studied in combination with Gemcitabine to overcome that resistance in clinical trials. For example, PDAC tumors frequently overexpress receptor tyrosine kinases such as Epidermal Growth Factor Receptor (EGFR) that activate downstream PI3K/AKT signaling to suppress Gemcitabine-induced apoptosis (168). The combination of Gemcitabine and the selective EGFR inhibitor Erlotinib was evaluated in a phase III randomized controlled trial, improving median overall survival by less than two weeks, leading to its approval by the FDA (169). A recent phase II trial showed that the addition of the EGFR-neutralizing antibody panitumumab (formerly ABX-EGF) to Gemcitabine and Erlotinib nearly doubled overall survival time when compared to Gemcitabine and Erlotinib alone (8.3 v 4.2 months), though this was associated with a higher incidence of grade 3 non-hematologic toxicities (82.6 *vs.* 52.2%) and no patient had a complete response (170).

Several other targeted therapies with sound basic supporting data have been combined with Gemcitabine, though none have shown a significant benefit beyond the marginal improvement seen with Erlotinib. Interestingly, despite sharing a similar mechanism of action to Erlotinib, the EGFR inhibitor Cetuximab failed to significantly improve survival (171). Additionally, as discussed previously, the Smoothened inhibitor Vismodegib also failed to improve outcomes when added to Gemcitabine in a clinical trial (165).

Vascular Endothelial Growth Factor (VEGF) overexpression is commonly seen in malignancies and is associated with a poor prognosis. Pancreatic cancer is generally avascular compared to other cancer types, though studies have shown a correlation with blood vessel density, tumor VEGF-A, and disease progression (172). Several studies have evaluated targeting VEGF receptors in combination with chemotherapy, including Bevacizumab and Axitinib, though neither combination showed benefit in a phase III trial despite promising phase II data (173, 174).

Additionally, high intratumoral expression of Hyaluronan (HA) in the pancreatic tumor microenvironment significantly impairs perfusion, thereby limiting drug delivery. In murine PDAC, Pegvorhyaluronidase Alfa (PEGPH20) degrades HA *in vivo*, increasing drug delivery. While a phase II trial combining Gemcitabine and PEGPH20 improved progression-free survival in patients with high HA tumor expression, there was a significant increase in thromboembolic events resulting in a clinical hold in the study and re-initiation with prophylactic Enoxaparin. The phase III trial was discontinued due to failure to improve overall survival (175). Interestingly, expression of Connective Tissue Growth Factor (CTGF) also correlates with fibrosis in PDAC. In preclinical models, the anti-CTGF antibody Pamrevlumab impaired fibrous tissue adhesion. Pamrevlumab was combined with Gemcitabine and Nab-Paclitaxel in a phase I/II study of locally advanced disease, which increased the rate of surgical resection in the study arm and improved survival irrespective of whether patients were resected. Based on these findings, the drug was granted Fast Track Designation and is currently enrolling in a phase III trial in advanced PDAC (176).

Additional studies have attempted to circumvent Gemcitabine resistance *via* the addition of another cytotoxic agent. As discussed, Gemcitabine is now used first-line in combination with Nab-Paclitaxel, which extended 2-year-survival from 4 to 9% in a clinical trial (10). The combination of Gemcitabine and Cisplatin is currently approved for patients with advanced PDAC and underlying *BRCA* mutations due to the excellent responses to platinum-based alkylating agents in tumors with deficient DNA repair mechanisms (177). This concept was expanded to include unknown DNA repair deficiencies that are likely present in all PDAC patients, and Cisplatin was added to the combination Gemcitabine and Nab-Paclitaxel in a phase I/II trial. Although the sample size was small (N=25), this combination was associated with significant improvement in response rates, including two patients with complete responses and a median overall survival of nearly double that of Gemcitabine and Nab-Paclitaxel alone. This multi-drug combination is now being investigated in other stages of the disease (178). However, *BRCA* mutations have long been suggested as a biomarker for sensitivity to PARP inhibitors such as Olaparib. This approach is based on the long-standing hypothesis that PARP inhibition will impair the repair of single-stranded breaks, causing synthetic lethality in tumors with loss of high-fidelity double-strand break homologous recombination (179). As Olaparib is now showing clear efficacy as maintenance therapy in *BRCA*-mutated PDAC (17), the addition of Olaparib to Gemcitabine and Cisplatin should be considered.

In addition to the above approaches, a European study has explored the combination of adjuvant Gemcitabine and Capecitabine. This combination extended median overall survival to 28 compared to 25.5 in the Gemcitabine group (122). However, earlier reports have not identified a significant difference between Gemcitabine with Capecitabine and Gemcitabine monotherapy. Hence, Capecitabine warrants continued exploration as a Gemcitabine adjuvant (180).

Emerging preclinical evidence suggests that long-term administration of Gemcitabine leads to extensive reprogramming of the pancreatic tumor microenvironment, enhancing a number of immune cell processes, including antigen presentation, PD-L1 expression, but promoting TGFβ biosynthesis which can act to suppress cancer-directed immune mechanisms. The combination of Gemcitabine, PD-1 inhibition, and TGFβ signal inhibition was highly effective in controlling disease in transgenic models of PDAC, far surpassing the efficacy of concomitant PD-1 and TGFβ signal inhibition without Gemcitabine (181, 182). Additionally, a recent preclinical study has identified signaling by the hepatocyte growth factor receptor MET as a potential driver of Gemcitabine resistance in PDAC. In this work, the authors show that MET is highly expressed at the plasma membrane of pancreatic cancer cells, and that TR1801-ADC, a novel antibody drug conjugate composed of a MET antibody conjugated to the highly potent pyrrolobenzodiazepine toxin-linker Tesirin was highly effective in MET-overexpressing patient derived xenografts, and synergized with Gemcitabine, even in tumors previously demonstrated to be resistant to Gemcitabine (183). Hence, these and other combination strategies warrant continued exploration in the treatment of Gemcitabine-refractory PDAC.

## Paclitaxel/Nab-Paclitaxel

**Figure 5:**
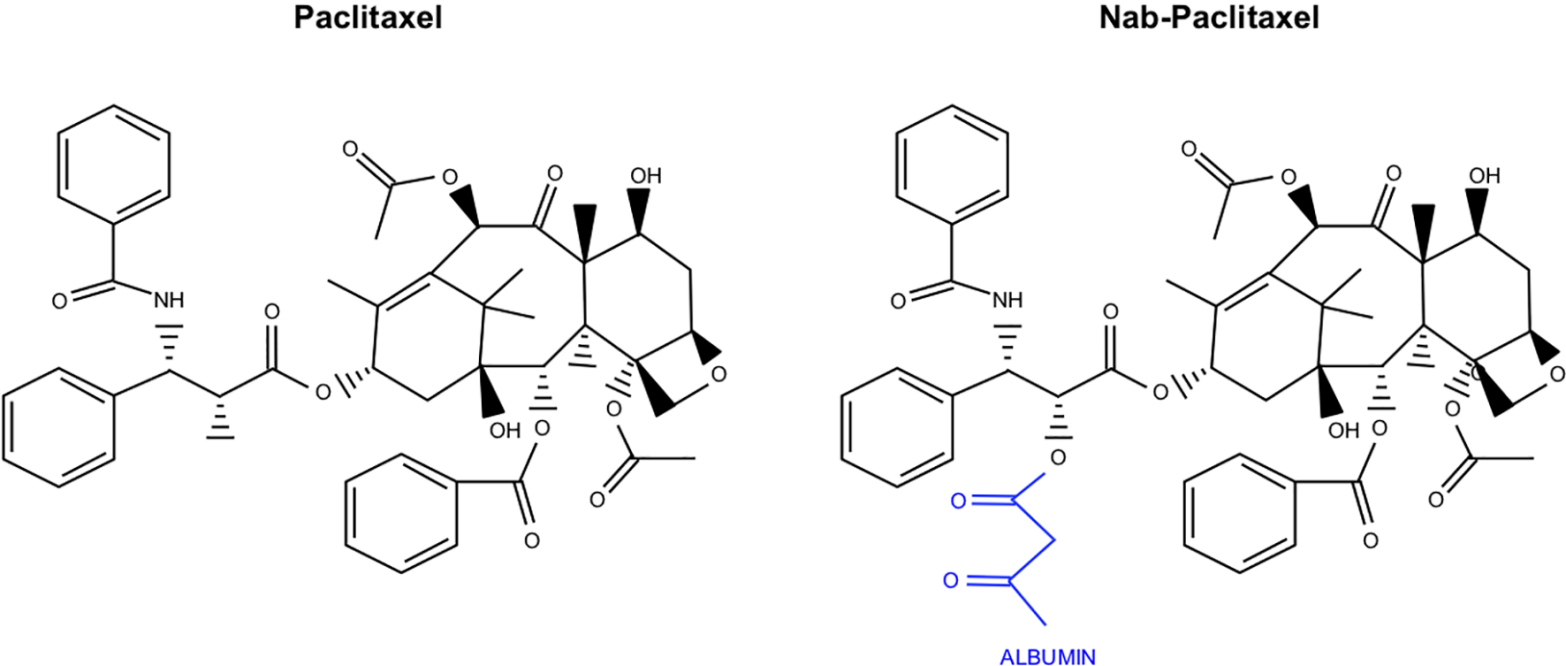
Chemical structure of Paclitaxel and the albumin conjugated variant Nab-Paclitaxel. Nab-Paclitaxel, used in combination with Gemcitabine in PDAC, varies from Paclitaxel in that it is conjugated to albumin as a delivery vehicle.

As part of a USDA initiative to identify natural compounds with therapeutic potential, researchers determined that Pacific yew tree bark extract had significant anti-neoplastic effects *in vitro*. Over the next five years, this sample was fractionated, and the most active component was identified. This compound was named Taxol and soon entered large scale biological testing. Taxol was then transferred from the USDA to Bristol-Myers Squibb for commercial development under the generic name Paclitaxel (**Figure 5**). Paclitaxel soon entered clinical evaluation in combination with Cisplatin, which was approved for use in ovarian cancer patients in 1992. Paclitaxel was next explored as monotherapy in breast cancer and was approved in patients with axillary node involvement in 1994. Paclitaxel was approved as a second-line treatment of AIDS-related Kaposi’s sarcoma in 1997 and in combination with Cisplatin for select cases of non-small cell lung cancer in 2006. Paclitaxel is also used off label to treat several other cancers, including those of the esophagus, stomach, endometrium, cervix, prostate, head and neck, as well as sarcomas, leukemias, and lymphomas (184).

While the initial formulation of Paclitaxel is still in clinical use, researchers soon identified that many of its adverse effects are caused by its solvent (185). This led to the conjugation of Paclitaxel to albumin (Nab-Paclitaxel), decreasing hydrophobicity and improving bioavailability (186) (**Figure 5**). This new formulation was approved for metastatic or refractory breast cancer in 2005, for non-small cell lung cancer in 2012, and for advanced pancreatic cancer in combination with Gemcitabine in 2013 (186–188). In pancreatic cancer, Nab-Paclitaxel is used exclusively. Compared to unconjugated Paclitaxel, Nab-Paclitaxel has increased solubility without many of the solvent associated toxicities. The albumin conjugate may have additional roles in facilitating transcytosis, namely by associating with the gp60 cell surface receptor, resulting in Caveolin-1 mediated invagination (189). Pharmacokinetic studies suggest that Nab-Paclitaxel has an improved volume of distribution and more rapid clearance than unconjugated Paclitaxel (190). Additionally, tumors generally have increased concentrations of Nab-Paclitaxel when compared with unconjugated Paclitaxel, suggesting the solvents used to deliver Paclitaxel may sequester the drug in micelles and inhibit its transport (191). Interestingly, the combination of Cremophor EL and dehydrated ethanol has been shown to inhibit the binding of Paclitaxel to albumin, suggesting that these solvents may hinder albumin-associated transport of Paclitaxel to the tumor microenvironment (190).

Regardless of entry, once inside the target cells, Paclitaxel appears to exert its antineoplastic effects in a concentration-dependent manner. *In vitro* experiments suggest that at low concentrations, Paclitaxel inhibits the formation of mitotic spindles without affecting the function of preformed spindles or arresting cells in mitosis (192). After prolonged treatment with low nanomolar concentrations, intracellular Paclitaxel will bind β-tubulin, thereby stabilizing the mitotic spindle. This prevents the segregation of chromatids, resulting in cells with multiple micronuclei that form around clusters of chromosomes (192). These cells are not arrested in mitosis but continue to S-phase where they undergo cytokinesis due to this extra DNA content (192). At higher concentrations, Paclitaxel associates with microtubules at the taxane-binding site. This induces their polymerization into stable bundles that are resistant to de-polymerization, arresting the cells in mitosis (193). While both of these mechanisms are cytotoxic, the mechanism observed at lower concentrations is more likely the main cause of Paclitaxel-induced cell death in human tumors, as these concentrations are more akin to those used in the clinic.

An added advantage of Nab-Paclitaxel in PDAC therapy may be due to its uptake by macrophages through a process called macropinocytosis, leading to activation of Toll-like receptor 4 (TLR4) pathways and M1 polarization of the macrophages that promotes their immunostimulatory potential (194). Importantly, in an *in vivo* orthotopic model using KPC pancreatic cancer cells in syngeneic C57BL/6 mice, Nab-Paclitaxel alone or in combination with Gemcitabine induced an increase in M1 macrophages within the orthotopic tumors (194).

### Paclitaxel Resistance

To date, there have been no mechanistic studies evaluating Nab-Paclitaxel resistance in PDAC, and clinical trial data is similarly limited. However, as Paclitaxel has been used for decades in the management of other malignancies, there are several studies in these tumor types that may offer insight into clinically useful strategies to augment the effects of Nab-Paclitaxel in PDAC. For instance, Multidrug Resistance Protein 1 (also known as MDR1, p-glycoprotein, ABCB1, or CD243) is a transmembrane ATP-dependent efflux pump. In the intestinal mucosa, MDR1 limits the oral bioavailability of Paclitaxel by promoting its export from epithelial cells (195). Several cancers, including PDAC, appear to have frequent overexpression of MDR1 or closely related genes, e.g., ABCC1/Multidrug resistance-associated protein 1 (MRP1), and their utility as predictive biomarkers warrants clinical investigation (195, 196).

Beyond its export, there are several potential mechanisms of Paclitaxel/Nab-Paclitaxel resistance that are directly related to the microtubules (197). Many Paclitaxel resistant cells possess alterations or mutations to β-Tubulin family members, which decreases the ability of the drug to stabilize microtubules (197–199). While this has yet to be thoroughly investigated in PDAC, a recent study suggests that βIII-Tubulin is strongly expressed in pancreatic cancer tissues and has important roles in tumor cell growth and survival (200). Further, silencing βIII-Tubulin enhanced the action of both Gemcitabine and Paclitaxel in PDAC cells (200). However, in ovarian cancer βII and βIII-Tubulin mutations do not appear to have a role in determining Paclitaxel sensitivity (201). Interestingly, βIVb-Tubulin inhibition appeared to sensitize PDAC Cells to the vinca alkaloids Vincristine, Vinorelbine, and Vinblastine, which also target the microtubules (202). In lung cancer cells, ERK-mediated upregulation of βIVb-Tubulin appears to promote Paclitaxel resistance (203). As there is a preclinical inhibitor (VERU-111) of βIII/βIV-tubulin that has already shown efficacy in PDAC xenografts (204), the addition of VERU-111 to Gemcitabine and Nab-Paclitaxel warrants consideration.

Several additional signaling events and cell processes have been linked to Paclitaxel resistance, particularly in breast cancer. In MCF7 cells, Paclitaxel resistance is associated with a function shift away from apoptosis and towards autophagic cell death (205). A similar study using the same cell line determined that overexpression of Bcl-XL inhibits the intrinsic pathway of programmed cell death, contributing to Paclitaxel resistant phenotypes (206). Interestingly, HER2/neu overexpressing breast cancer cells appear to have diminished responses to Paclitaxel (207). While HER2 is overexpressed in a small subset of PDAC patients (208), the HER2-inhibiting antibody Trastuzumab failed to improve outcomes in HER2 overexpressing PDAC patients (209). Hence, while HER2 may be a poor target for therapy, HER2 may be a useful biomarker in determining Nab-Paclitaxel sensitivity.

Beyond the well-characterized role of aberrant apoptotic and autophagy-associated signals (210–214), several other cellular processes also appear to contribute to Paclitaxel responses. These include hypoxia, which induces mTOR-mediated autophagy and concurrent JNK-mediated pro-survival signaling (215). Additionally, overexpression of the centrosomal ninein-like protein (NLP) also appears to impede Paclitaxel-induced cell death (215). Finally, there is a growing body of evidence suggesting that miRNA dysregulation may also have important roles in Paclitaxel responses. Overexpression of miR100 sensitized MCF7 cells to Paclitaxel-induced cell death in part by targeting mTOR (216). Similarly, overexpression of miR-16 and miR-17 synergized to reduce Bcl2 and autophagy, respectively, sensitizing tumor cells to Paclitaxel *in vitro* (217). These mechanisms are largely uncharacterized in PDAC and should be carefully explored to identify those most pertinent to therapy.

## 5-Fluorouracil

**Figure 6:**
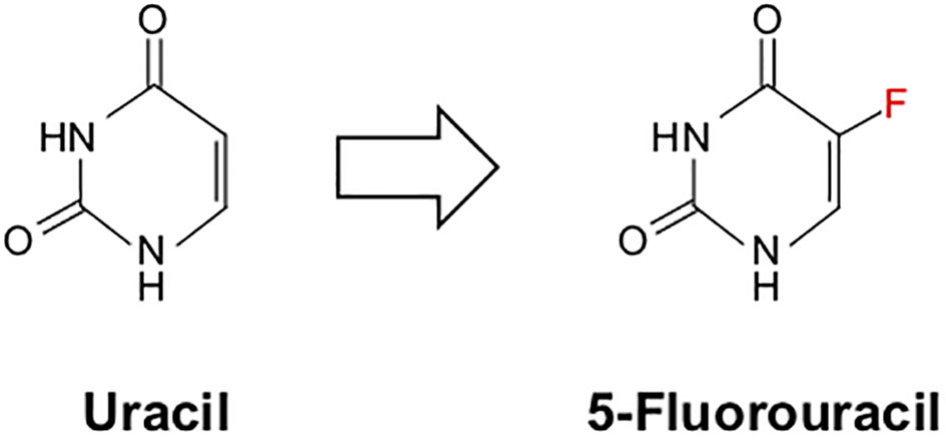
Chemical structure of uracil and its chemical mimic 5-Fluorouracil. 5-Fluorouracil (5-FU) is a nucleoside analog identical in structure to uracil apart from a single fluoride molecule at the 5’-position.

Initially patented in 1956, 5-FU has been a mainstay in cancer treatment since its FDA approval in 1962. 5-FU is currently used in several cancers, including breast, colon, esophagus, stomach, and pancreatic cancer, as part of the multidrug regimen FOLFIRINOX. In 1957, the Heidelberger group at the University of Wisconsin reported that rat hepatoma cells displayed increased uptake of Uracil when compared to normal cells (218). Based on these observations, they synthesized a modified Uracil with a fluoride substitution at the 5-position, fluoropyrimidine 5-fluorouracil or 5-FU (218) (**Figure 6**). This compound was wildly successful, showing anti-neoplastic activity against various cancer cells, and was immediately introduced to the clinic before obtaining a patent.

In their 1958 trial involving 103 cases of solid and hematologic malignancies, this group observed an approximate 25% response rate, though regression was only seen in those with severe toxicity (219). These observations, combined with the lack of 5-FU resistance seen in this cohort, eventually led to the addition of 5-FU to cyclophosphamide, methotrexate, and prednisone in 1972 to form the CMFP combination regiment for metastatic breast cancer. This combined approach had a complete remission rate of 20% and a partial remission rate of 40%. By omitting prednisone to create the CMF regimen still used today, physicians observed response of 57%. These data led to several additional studies centered on 5-FU, which would eventually be approved for the many other cancer types described, including pancreatic cancer, as part of the FOLFIRINOX regimen. As discussed, this approach showed improved efficacy when compared to single agent Gemcitabine in metastatic PDAC (220), and as a result, FOLFIRINOX was soon approved and is now a first-line treatment.

**Figure 7:**
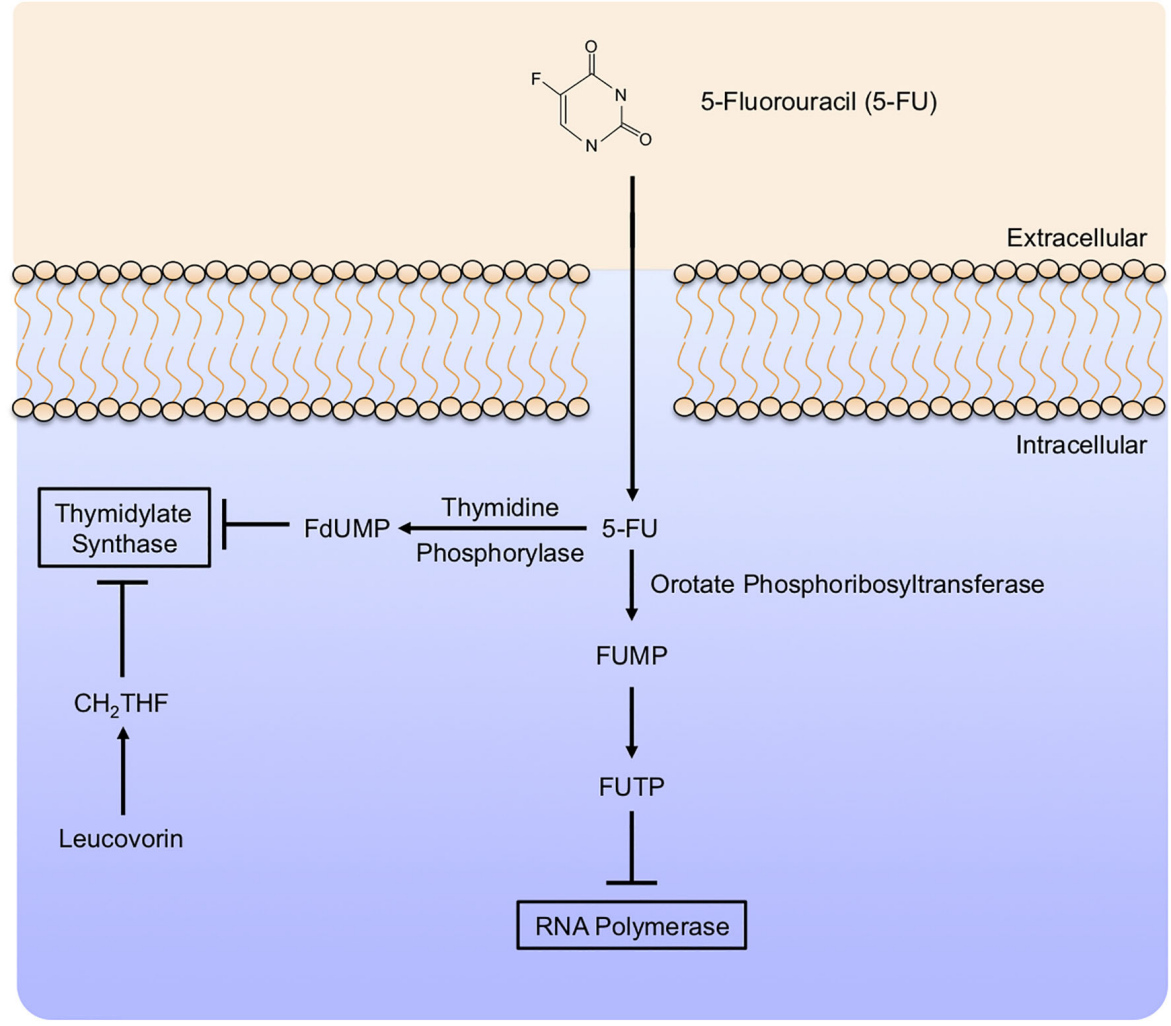
5-Fluorouracil mechanism of action. 5-Fluorouracil (5-FU) enters target cells in a similar manner to uracil, mainly by facilitated transport. Like Gemcitabine, 5-Fluorouracil (5-FU) is modified extensively in the cytoplasm. This includes conversion to Fluorodeoxyuridine monophosphate (FdUMP) by Thymidine Phosphorylase, inhibiting Thymidylate Synthase and potentiated by the addition of Leucovorin. In a parallel mechanism, 5-FU can be further modified to Fluorodeoxyuridine triphosphate, which subsequently inhibits RNA Polymerase.

5-FU appears to have a variety of effects in the target cells, many of which are not entirely clear. 5-FU is taken into cells in the same manner as Uracil, both sharing a saturable, facilitated transport mechanism (221). Similar to Gemcitabine, intracellular 5-FU interacts with a variety of modifying enzymes, converting it to several active metabolites. These include Fluorodeoxyuridine monophosphate (FdUMP), Fluorodeoxyuridine triphosphate (FdUTP), and Fluorouridine triphosphate (FUTP), many of which are responsible for the anti-neoplastic effects of 5-FU, mainly *via* the inhibition of Thymidylate Synthase (**Figure 7**).

5-FU may have tumoricidal effects extending beyond Thymidylate Synthase inhibition, though these are less clear. 5-FU appears to have additional effects on transcription, namely *via* incorporating its metabolite FUTP into RNA (222). The incorporation of 5-FU metabolites into RNA is itself cytotoxic, suppressing the growth of both breast (223) and colon (224) cancer cells. 5-FU also appears to interfere with the maturation processing of both mRNA and tRNAs through various alternate mechanisms, all of which appear to further dysregulate critical cell processes and reduce viability (222).

### 5-FU Resistance

Despite being used in cancer treatment for nearly 60 years, there is little known regarding 5-FU resistance, particularly in PDAC. While some studies suggest that PDAC cells acquire a means to resist 5-FU through altered expression of select apoptotic regulators, or aberrant cell processes, including autophagy, none has translated into a successful adjuvant therapy for pancreatic cancer patients (225, 226). The majority of clinical studies exploring 5-FU have been conducted in colon cancer. As single-agent response rates are low (227), investigators have long been exploring combination strategies to enhance the efficacy of 5-FU based chemotherapy. The addition of other anti-cancer agents to 5-FU has helped to achieve response rates as high as 80% (228), and this approach is now the standard-of-care in several tumor types. However, these approaches often predispose patients to additive toxicities. Therefore, like the other drugs described in this review, there is a need to better understand the mechanisms through which tumor cells resist 5-FU in hopes that these events can be reversed, and drug responses can be achieved without the use of higher doses or additional chemotherapies.

As mentioned, no studies have identified a precise, clinically actionable mechanism of 5-FU resistance in PDAC. However, several studies in other cancers have suggested that several aberrant cell processes can limit the anti-tumor efficacy of 5-FU in other cancers (229). For instance, in a study of 13 colon cancer cell lines, though there was no predictive mechanism with respect to drug transport, accumulation, or Thymidylate Synthase kinetics, increased expression of Thymidylate Synthase was strongly associated with reduced 5-FU sensitivity (230). Accordingly, the induction of Thymidylate Synthase has similarly been shown to impede 5-FU responses *in vitro*, and in patients, Thymidylate Synthase expression was directly related to the efficacy of 5-FU (230). Hence, Thymidylate Synthase inhibition has long been suggested as a potential means of overcoming 5-FU resistance (230–232).

Several additional mechanisms of 5-FU resistance have also been suggested and have been reviewed extensively (229). Notably, cells with diminished P53 expression had increased 5-FU sensitivity, suggestive of a role for defective DNA repair processes in mediating drug responses (230). Should this be true of PDAC tumors, this may be of some clinical utility given the high frequency of *TP53* mutations. Other recent evidence appears to implicate WNT signaling in 5-FU resistance, namely *via* suppressing the checkpoint kinase 1 (CHK1) pathway, particularly in P53 wild-type cells (233). Hence, these and other mechanisms must be exhaustively characterized in PDAC to further enhance the therapeutic efficacy of the FOLFIRINOX regimen.

## Irinotecan

Like Paclitaxel, the commercial development of Irinotecan can be traced back to a screen of natural products (234). In 1966, investigators isolated Camptothecin from the bark/stem of the *Camptotheca acuminate* or Happy tree. This tree is indigenous to China, and its extract had long been used in traditional Chinese medicine as an anti-cancer agent (234). This compound was found to have significant anti-tumor activity but was associated with severe toxicity in preclinical testing (235). In an effort to develop a similar compound with a more favorable adverse effect profile, the water-soluble Camptothecin analog Irinotecan was synthesized. While Irinotecan demonstrated little anti-tumor activity *in vitro*, its metabolite 7-Ethyl-10-Hydroxycamptothecin (SN-38) displayed up to a 2000-fold increase in potency (236, 237). Irinotecan soon entered clinical trial and was approved by the FDA as a second-line monotherapy for metastatic colorectal cancer in 1996 (238). In a subsequent trial, the addition of Irinotecan to 5-FU/Leucovorin resulted in twice the response rate compared to 5-FU/Leucovorin monotherapy, resulting in the approval of Irinotecan as first-line therapy in 1998. Irinotecan was approved for metastatic PDAC in 2013, where it is currently used as part of the multidrug regimen FOLFIRINOX (239).

**Figure 8:**
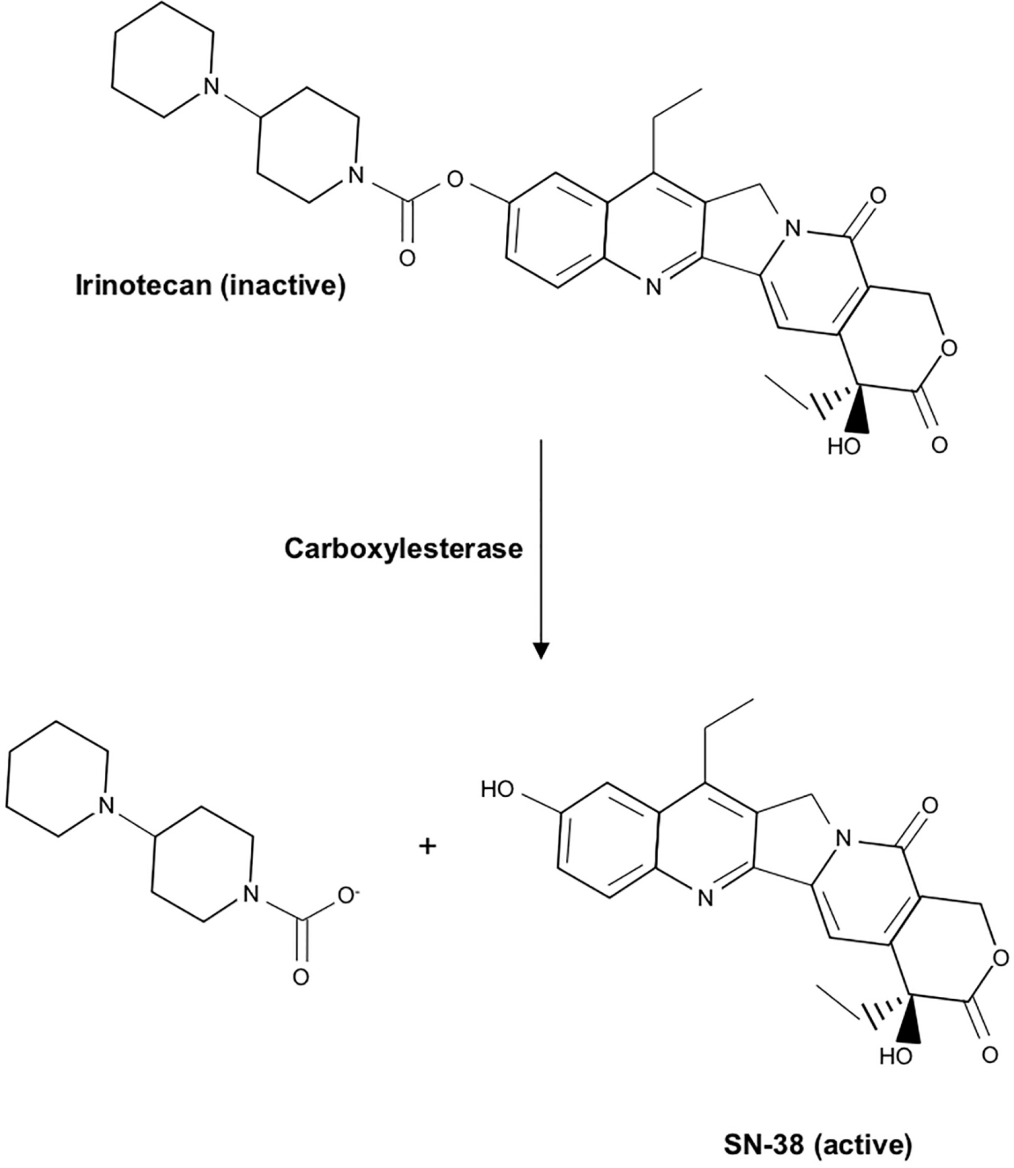
Chemical structure of Irinotecan and conversion to its active metabolite SN-38. In the intestinal mucosa, liver, and plasma, Irinotecan undergoes carboxylesterase-mediated cleavage of the carbamate bond between the camptothecin moiety and the dipiperidino side chain, converting water-soluble Irinotecan to lipid-soluble SN-38, its active metabolite.

Like Camptothecin, Irinotecan functions as a topoisomerase inhibitor that functions by primarily impeding DNA synthesis during S-phase. Structurally, the two are quite similar, containing a pentacyclic ring structure with a pyrrole (3, 4 β) quinoline moiety, an S-configured lactone form, and a carboxylate form. However, Irinotecan has an additional ethyl group, and a bipiperidinocarbonyloxy group in place of two hydrogen molecules, improving water solubility and reducing many of the severe toxicities observed in response to Camptothecin. In the intestinal mucosa, liver, and plasma, Irinotecan undergoes extensive modification, namely carboxylesterase-mediated cleavage of the carbamate bond between the camptothecin moiety and the dipiperidino side chain. This converts water-soluble Irinotecan to lipid-soluble SN-38 (**Figure 8**). SN-38 is roughly 1000 times more potent than Irinotecan, and is believed to be responsible for most anti-tumor effects (240).

The mechanisms through which SN-38 induces cell death have been well described. During DNA replication, Topoisomerase I introduces a single strand knick into the DNA backbone in order to relieve tension (235). While these strands would normally be rejoined later in the DNA replication process, SN-38 stabilizes the association of Topoisomerase I to the 3’-phosphate at the DNA break site. This inhibits the ligating function of Topoisomerase I, resulting in persistent single-strand DNA breaks (235). These breaks interfere with further DNA synthesis, causing double-strand DNA breaks, the arrest of the S-phase, and apoptosis (235).

### Irinotecan Resistance

Like many other medications described in this review, there is little data available regarding Irinotecan resistance in PDAC. Like 5-FU, Irinotecan has been used longer in the clinical management of colon cancer. Hence, there are more insights into Irinotecan resistance in colon cancer cells, though none has translated into a successful adjuvant therapy at this time. In colon cancers, Irinotecan resistance appears to be mediated through several mechanisms, including reduced intracellular concentrations of SN-38. This is often mediated by active transport of either Irinotecan of SN-38 by the ATP-binding cassette gene multidrug resistance protein or MRP (241). However, several other factors appear to have driving roles in Irinotecan resistance, including variable levels of converting, reduced Topoisomerase I expression, Topoisomerase I mutation, alterations in the cellular response to Camptothecin-Topoisoomerase I-DNA complex formation, and the activation of multiple signaling cascades including NFκB, EGFR, and SRC (242, 243). However, these are almost entirely unexplored in pancreatic cancer and warrants further evaluation given the advent of the FOLFIRINOX regimen.

## Oxaliplatin

Platinum-based agents have been used successfully utilized in cancer therapy for decades. The first platinum-based anti-neoplastic Cisplatin was synthesized in 1845, though it was not approved for cancer patients until 1978 (244). In the decades since, Cisplatin has been used in several cancer types and remains one of the most widely used anti-cancer medications in the developed world. Its success led to the discovery of several similar agents, including Oxaliplatin. Stemming from efforts to improve the toxicity, solubility, and resistance profiles of Cisplatin, Oxaliplatin was first approved for clinical use in Europe in 1996, and the first New Drug Application (NDA) for Oxaliplatin was submitted to the FDA in 1999.

In early trials, while Oxaliplatin demonstrated clear anti-tumor activity in colorectal cancer, it failed to show an increased survival benefit compared to the then standard-of-care treatment, and it was not approved for use. However, subsequent trials examining the combination of Oxaliplatin, 5-FU/Leucovorin, and Irinotecan in metastatic colon cancer showed that the combination had an increased response rate compared to either Oxaliplatin or 5-FU monotherapy and offered a 2-month increase in progression-free survival (245). This led to 2002 FDA approval of the combined regimen in metastatic colorectal cancers that had either recurred or progressed during or within six months of completion of first-line therapy (246). Oxaliplatin has since been approved for use in metastatic PDAC, where it is currently used as part of the multidrug regimen FOLFIRINOX.

**Figure 9:**
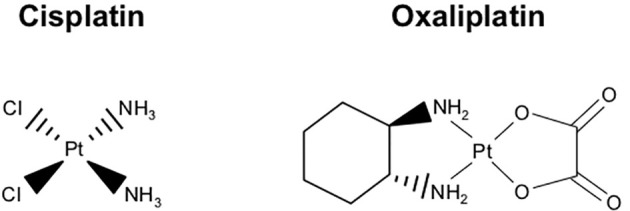
Chemical structure of Oxaliplatin and structural similarity to Cisplatin. Oxaliplatin is structurally similar to Cisplatin, though it differs in the replacement of amine groups with diaminocyclohexane and the chlorine ligands with oxalate, thereby increasing antitumor activity and water solubility.

Oxaliplatin is structurally similar to Cisplatin, differing in the replacement of amine groups with diaminocyclohexane (DACH) and the chlorine ligands with oxalate (247) (**Figure 9**). These structural modifications have been suggested to both increase antitumor activity and water solubility (248). Oxaliplatin is thought to enter tumor cells through a variety of transporters, namely cation transporters hOCT2 and hOCT3 (249, 250). Once inside the target cells, Oxaliplatin is converted to its active derivatives *via* several successive reactions, including non-enzymatic displacement of the oxalate ligand, its replacement with chloride ions, and subsequent hydrolysis. These transient reactive species bind macromolecules, specifically neighboring guanine moieties of DNA, resulting in intrastrand crosslinks (248). The current consensus is that Oxaliplatin-mediated cytotoxicity directly results from the formation of these DNA crosslinks, and the subsequent effects on DNA replication and transcription (251). Interestingly, Oxaliplatin has also been shown to initiate interstrand cross-links at a lower frequency, which may also have a role in its tumoricidal activity (251).

### Oxaliplatin Resistance

Resistance to Oxaliplatin has been extensively evaluated in several cancers and appears to be largely mediated by a variety of transport proteins. For instance, overexpression of multidrug resistance-associated protein 1 (MRP1), a unidirectional efflux transporter also known as ABCC1, is associated with decreased drug accumulation and increased resistance in ovarian cancer cells (252). Additional studies suggest that glutathione-mediated export may also contribute to Oxaliplatin resistance, though this is less established and likely context-specific. For instance, select studies have determined that increased levels of glutathione are associated with poor responses Oxaliplatin (253); however, others have found no association between glutathione and Oxaliplatin or even a positive association between glutathione levels and Oxaliplatin sensitivity (254, 255). Hence, these and other export mechanism warrants continued exploration, particularly in PDAC.

Several other additional resistance mechanisms have also been identified. For instance, in addition to diminished drug uptake, several Oxaliplatin-resistant cell lines demonstrate decreased platinum accumulation in their DNA and increased tolerance of DNA adducts (256). This has been suggested to stem from an increased propensity for base excision repair (BER), as colorectal cancers with low expression of the BER mediator excision repair cross-complementing (ERCC1) have been shown to have increased sensitivity to Oxaliplatin (257). Other potential resistance mechanisms appear to stem from alterations to the apoptosis program. Potential mediators include pyruvate kinase isoform M2, or loss of Bax-mediated apoptosis, though these have yet to be fully characterized in PDAC (258–261).

## Summary

There is currently no effective treatment for PDAC. Given the lack of early detection or chemoprevention strategies, most patients will present with advanced disease. For those with operable cancer, surgery can significantly extend survival, though overall outcomes remain poor. As discussed, there are many factors limiting the efficacy of both surgical and medical treatments of PDAC. While alternate approaches such as immunotherapy are beginning to show promise in clinical trials, there is an urgent need to better understand the limitations of the current standard of care approach for PDAC. With respect to chemoresistance, this is largely unexplored in PDAC, particularly for non-Gemcitabine agents. Thus, it is essential to improve our collective understanding of the mechanisms that underlie the near-universally poor therapeutic responses seen in PDAC patients to enhance drug efficacy, extending patient survival, and improve quality of life.

## Author Contributions

DP and PU drafted the manuscript. DP assembled figures. MK, JT, HM, and AR edited the manuscript. All authors contributed to the article and approved the submitted version.

## Funding

This work was supported by Veterans Affairs Merit Award I01BX004903 and Career Scientist Award IK6 BX004855 to AR, by NIH R01CA07059 to M. Korc, by NIH F30CA236031 and UIC Award for Graduate Research to DP, by NIH R01CA242003 and the Joseph and Ann Matella Fund for Pancreatic Cancer Research to JT, and by NIH R01CA217907, NIH R21CA255291 and Veterans Affairs Merit Award I01BX002922 to HM.

## Disclaimer

Per the funding policy of the U.S. Department of Veterans Affairs, we are required to state that these contents do not represent the views of the U.S. Department of Veterans Affairs or the United States Government.

## Conflict of Interest

The authors declare that the research was conducted in the absence of any commercial or financial relationships that could be construed as a potential conflict of interest.
